# Basal and stress-induced Hsp70 are modulated by ataxin-3

**DOI:** 10.1007/s12192-012-0346-2

**Published:** 2012-07-10

**Authors:** Christopher P. Reina, Barzin Y. Nabet, Peter D. Young, Randall N. Pittman

**Affiliations:** 1 Department of Pharmacology, University of Pennsylvania School of Medicine, Philadelphia, PA 19104 USA; 2 Present Address: Department of Molecular Biology and Biochemistry, Rutgers University, Piscataway, NJ 08854 USA; 3 Present Address: Department of Cancer Biology, University of Pennsylvania School of Medicine, Philadelphia, PA 19104 USA

**Keywords:** Hsp70 regulation, Hsf1, Homeostasis, Proteostasis, Stress, SCA3/MJD

## Abstract

Regulation of basal and induced levels of hsp70 is critical for cellular homeostasis. Ataxin-3 is a deubiquitinase with several cellular functions including transcriptional regulation and maintenance of protein homeostasis. While investigating potential roles of ataxin-3 in response to cellular stress, it appeared that ataxin-3 regulated hsp70. Basal levels of hsp70 were lower in ataxin-3 knockout (KO) mouse brain from 2 to 63 weeks of age and hsp70 was also lower in fibroblasts from ataxin-3 KO mice. Transfecting KO cells with ataxin-3 rescued basal levels of hsp70 protein. Western blots of representative chaperones including hsp110, hsp90, hsp70, hsc70, hsp60, hsp40/hdj2, and hsp25 indicated that only hsp70 was appreciably altered in KO fibroblasts and KO mouse brain. Turnover of hsp70 protein was similar in wild-type (WT) and KO cells; however, basal hsp70 promoter reporter activity was decreased in ataxin-3 KO cells. Transfecting ataxin-3 restored hsp70 basal promoter activity in KO fibroblasts to levels of promoter activity in WT cells; however, mutations that inactivated deubiquitinase activity or the ubiquitin interacting motifs did not restore full activity to hsp70 basal promoter activity. Hsp70 protein and promoter activity were higher in WT compared to KO cells exposed to heat shock and azetidine-2-carboxylic acid, but WT and KO cells had similar levels in response to cadmium. Heat shock factor-1 had decreased levels and increased turnover in ataxin-3 KO fibroblasts. Data in this study are consistent with ataxin-3 regulating basal level of hsp70 as well as modulating hsp70 in response to a subset of cellular stresses.

## Introduction

Hsp70^1^ is a key protein in cellular homeostasis and increases rapidly from a low basal level to high levels in response to a variety of stresses. In response to cellular stress, heat shock factor 1 (Hsf1) is activated and initiates a sequence of events including robust activation of the hsp70 promoter. The increased level of hsp70 protein protects stressed cells by binding and processing unfolded, misfolded, and aberrant proteins (Lindquist and Craig 1988; Parsell and Linquist 1993; Hartl et al. 1994; Voellmy 1994; Wu 1995; Morimoto 1998).

Under normal conditions, most cells and tissues have low levels of hsp70. Maintaining the basal level of hsp70 is important for normal cellular functions (Feder et al. 1992; Volloch and Sherman 1999; Hartl and Hayer-Hartl 2002). Basal hsp70 appears to regulate several critical cellular functions including protein folding and transport (Hartl et al. 1994; Hartl and Hayer-Hartl 2002), helping maintain Hsf1 in an inactive state (Abravaya et al. 1992; Mosser et al. 1993; Rabindran et al. 1994; Morimoto 1998), regulating apoptosis (Beere and Green 2001), and cell proliferation (Milarski and Morimoto 1986; Milarski et al. 1989; Taira et al. 1999). Basal hsp70 has several critical functions; however, only a few proteins have been identified that regulate basal levels of hsp70.

Ataxin-3 (Atxn3) is a deubiquitinase (DUB; Burnett et al. 2003; Scheel et al. 2003) present in both plants and animals and expressed in all or most tissues in humans and mice. Expansion of a polyglutamine tract in Atxn3 results in the fatal neurodegenerative disease, spinocerebellar ataxia type 3 (SCA3; also known as Machado–Joseph disease (MJD); Kawaguchi et al. 1994). SCA3/MJD is a protein conformational disease (proteinopathy) along with many other neurodegenerative diseases including nine polyglutamine diseases, Alzheimer’s disease, Parkinson’s disease, amyotrophic lateral sclerosis, prion diseases, and additional diseases that are defined by accumulation of misfolded proteins and/or accumulation of proteins with altered conformations (Carrell and Lomas 1997; Bucciantini et al. 2002; Walsh et al. 2002; Sanchez et al. 2003). While pathogenic Atxn3 is responsible for the protein conformational disease, SCA3/MJD, normal wild-type Atxn3 has multiple cellular functions, including helping maintain protein homeostasis (Matos et al. 2011). Processes regulated by Atxn3 in protein homeostasis include editing ubiquitinated proteins (Burnett et al. 2003; Schmitt et al. 2007; Winborn et al. 2008; Todi et al. 2009; Kuhlbrodt et al. 2011), sequestering aggregated proteins in aggresomes (Burnett and Pittman 2005; Ouyang et al. 2012), modulating E3 ubiquitin ligases (Durcan et al. 2010; Scaglione et al. 2011), protein degradation (Burnett et al. 2003; Doss-Pepe et al. 2003; Zhong and Pittman 2006; Wang et al. 2006; Schmitt et al. 2007), and stress response (Reina et al. 2010; Araujo et al. 2011; Rodrigues et al. 2011).

Recent work in our lab demonstrated that Atxn3 responded to select proteotoxic stresses by altering its interactions with two shuttle proteins that function in protein degradation, valosin-containing protein (VCP/p97) and human Rad23 (HR23B); furthermore, in response to heat shock and oxidative stress Atxn3 translocated to the nucleus (Reina et al. 2010). In follow up experiments, characterizing Atxn3 functions in response to cellular stress, we observed that basal levels of hsp70 appeared to be lower in Atxn3 KO cells. Hsp70 regulation and its functions are critical for cellular homeostasis; therefore, we examined the possibility that Atxn3 modulates hsp70.

## Materials and methods

### Cell culture and treatments

Primary fibroblasts were isolated from postnatal day 4 WT and Atxn3 KO mouse skin. Several independent primary fibroblasts cultures were maintained continuously and subcultured as needed until cells emerged that had strong proliferation properties. Lines of WT and KO fibroblasts were established by multiple subcultures.

WT and KO fibroblasts were exposed to stressors that induce hsp70 including heat shock, cadmium, and azetidine-2-carboxylic acid (AZE), as well as 2-deoxyglucose (2-DG), a stressor that does not routinely induce hsp70. Preliminary studies identified concentrations and times that minimized toxicity and resulted in appreciable hsp70 promoter activation (or >80 % decrease in ATP in the case of 2-DG). Conditions used for stressors in the study were the following: (1) tissue culture dishes were “sealed” with parafilm and heat shocked at 42 °C in a water bath for 30 or 45 min; times were restricted to a maximum of 45 min due to heat-induced misfolding of firefly luciferase and *Renilla* luciferase (Harrison et al. 2006); (2) cadmium chloride at 50 uM for 2–5 h; (3) AZE at 2.5 mM for 6–18 h; and (4) 2-DG at 10 mM for 1–3 h. WT and KO fibroblasts were also treated with 10 uM cycloheximide (Sigma) 0–8 h to determine turnover of hsp70 and Hsf1 and treated with 5 uM of the irreversible proteasome inhibitor, clasto-lactacystin-*β*-lactone (Biomol), for 1–4 h to follow accumulation of Hsf1.

### Cell sorting

Atxn3 KO fibroblasts (600,000 cells/100 mm dish) were plated and transfected 24 h later with Lipofectamine 2000 (60 ul; Invitrogen) and 24 ug of either GFP or GFP-Atxn3Q22. After 24 h of transfection, cells were removed from dishes with trypsin/EDTA, triturated in DMEM containing 10 % serum, counted, pelleted in a centrifuge, and pellets suspended at a concentration of 5 × 10^6^/ml in Dulbecco’s phosphate-buffered saline (PBS; without magnesium chloride or calcium chloride) + 2 % BSA and 25 mM HEPES, pH 7.55. Each cell sorting experiment consisted of GFP transfected cells and GFP-Atxn3 transfected cells; presorting processing was staggered to decrease cell aggregation. Just prior to sorting cells, they were passed through a 35 um mesh to decrease aggregates. Cells were sorted using a FACSVantage SE cell sorter and FACSDiVa software by the staff in the Flow Cytometry and Cell Sorting Facility (University of Pennsylvania). Immediately following the sort, cells were pelleted and frozen at −80 °C until used for western blots.

### Western blots and antibodies

Cultured fibroblasts were rinsed with PBS, scraped from dishes in PBS, pelleted in a centrifuge, and processed for western blots or stored at −80 °C. Cell pellets were solubilized in 1 % Triton X-100 in PBS and sonicated with twelve 1-s pulses at level 3.5 (Sonic Dismembrator 60, Fisher Scientific). An aliquot was removed for a protein assay and 4× sodium dodecyl sulfate (SDS) sample buffer added to the remaining sample to generate a final lysate of 1× SDS sample buffer. Whole mouse brains were removed and placed in 5 ml of ice-cold PBS and disrupted using a Polytron (Brinkmann Instruments) on setting 5 for 15 s. Lysate was removed (1.6 mls) and NP40 added to make a final concentration of 0.1 %. The sample was triturated for 20 s, sonicated with twenty 1-s pulses at level 5.5 and centrifuged at 20,000×*g* for 20 min. An aliquot was removed for protein assay and 4× SDS sample buffer added to the remaining lysate to generate a final lysate of 1× SDS sample buffer. Samples were 70 ug protein for cell lysates and 100 ug protein for brain tissue lysates unless indicated otherwise.

Following electrophoresis, gels were transferred to PVDF membranes and blots incubated with the following antibodies at the indicated concentrations: Ataxin-3 1 H9 (1:20,000; Millipore MAB5360), glyceraldehyde 3-phosphate dehydrogenase (GAPDH; 1:40,000; Enzo CSA-335), HSC-70 (1:2,500; Enzo SPA-815), HSP-25 (1:1,000; Enzo SPA-801), HSP-40 Hdj2 (1:1,000; Enzo SPA-405), HSP-60 (1:3,500; GeneTex GTX110089), HSP-70 (1:2,000; Enzo SPA-810), HSP-90 (1:1,000; Enzo SPA-846), HSP-110 (1:1,000; Enzo SPA-1101), Hsf1 (1:1,000; Enzo SPA-950). Western blots were processed with antibodies and then treated with Western Lighting^®^Plus-ECL (PerkinElmer) chemiluminescence reagent and exposed to HyperFilm™ ECL (Amersham). In many cases, western blots were stripped using Restore™ stripping buffer (Thermo Scientific) and then reprobed with other antibodies.

For quantitation of western blots, films were scanned at a resolution of 600 dpi. The area of interest was cropped and saved as a TIFF file and analyzed with ImageJ software. Individual boxes were created in ImageJ to encompass each band; the density of each band and background was quantitated and the background subtracted. For most statistical comparisons, the quantitated bands were normalized to GAPDH from the same sample and lane (blots were reprobed with GAPDH so that each lane and band could be normalized). A two-tailed student’s *t* test was used to determine significance.

### Hsp70 ELISA assay

Whole brains were removed from WT and Atxn3 KO mice aged 2, 4, 6, 26, or 63 weeks old. Brains were split along the median to generate two samples. Half brains were snap-frozen in liquid nitrogen, weighed (range, 150–250 mg/half brain) and stored at −80 °C. Frozen brain samples were thawed on ice and 150 μl of 10 mM Tris buffer added. Samples were homogenized with a tissue homogenizer (Omni International, TH2000) for 15 s followed by adding 50 μl of 5× extraction buffer from the Hsp70 EIA Kit (Enzo Life Sciences, EDKS-700B). Following a 30-min incubation on ice, samples were sonicated with 12 pulses on level 7 (Sonic Dismembrator 60, Fisher Scientific) and then centrifuged at 20,000×*g* for 5 min. The supernatant was diluted 1:5 in sample dilutent 2 (component in Hsp70 EIA Kit); 100 ul duplicates were used in the enzyme-linked immunosorbent assay (ELISA) assay and the remaining supernatant was used for a protein assay and excess supernatant was stored at −80 °C. Each 96-well ELISA plate (Hsp70 EIA Kit) contained duplicates of brain samples (1–2 mg protein), control lysate (2.5 mg protein), and hsp70 standards (1.25, 0.625, 0.313, 0.156, 0.078, 0.039, 0.02, and 0 ng). Control lysate was a mixture of multiple different lysates; aliquots of control lysate were frozen at −80 °C and used to normalize brain samples in each experiment. The protocol provided with the Hsp70 EIA Kit was followed without modifications for quantification of Hsp70 protein levels. A Multiskan Plus (Thermo Scientific) was used to measure absorbance at 450 nm and the data was analyzed using Ascent for Multiskan and Microsoft Excel. A two-tailed student’s *t* test was used to determine significance.

### Luciferase assay

Fibroblasts were plated in 24-well dishes at concentrations to generate cell densities 24 h later at 40–50 % confluence (for 48 h transfections) or at 70–80 % confluence (for 24 h transfections). Cells were transfected using the protocol provided with Lipofectamine 2000 (Invitrogen) for NIH3T3 cells. The transfection was optimized with Lipofectamine 2000 at a ratio of 2:1 to DNA. Total amounts of DNA were optimized for 24 and 48 h transfections for expression and low toxicity: (1) 24-h transfections: (a) used to compare WT and KO cells— 300 ng Hsp70-luc (luc = firefly luciferase), 10 ng *Renilla* luciferase (Promega); (b) used to compare effects of GFP vs GFP-Atxn3 in KO cells—300 ng Hsp70-luc, 10 ng *Renilla* luciferase, 300 ng GFP or GFP-Atxn3; (2) 48-h transfections: (a) used for WT and KO cells treated with AZE—300 ng Hsp-luc, 10 ng *Renilla* luciferase; (b) used to compare effects of GFP vs GFP-Atxn3 in KO cells treated with AZE–150 ng Hsp-luc, 5 ng *Renilla* luciferase, 150 ng GFP, or GFP-Atxn3. *Renilla* luciferase was included as an internal control reporter.

After 24–48 h of transfection, firefly and *Renilla* luciferases were quantitated using the protocol and kit for Dual-Luciferase Reporter Assay System (Promega) without modifications. Cells were washed three times with PBS and lysed in 100 ul passive lysis buffer (Promega); routinely, 20 ul of cell lysate was used for the assay. Both firefly luciferase activity and *Renilla* luciferase activity were measured with a TD20/20 Luminometer (Turner Designs) and analyzed using Microsoft Excel. The ratio of firefly luciferase (hsp70 promoter activity) to *Renilla* luciferase (internal control) was determined for each well and triplicates of each condition were averaged. Each experiment was repeated three to five times over a period of several weeks to insure that observations were consistent over time. A two-tailed student’s *t* test was used to determine significance.

### Knockout mice

Atxn3 knockout mice were generated at Lexicon Genetics from Omnibank ES cells containing a gene trap insertion in the Atxn3 gene. The gene trap vector, VICTR48, was inserted in the first intron downstream of the ATG; the intron sequence flanking the insertion site (*) is the following: 5′TCAAGTCATTTGGGTGTTTCTCGGACAA*CCATGTTTCATAATCATTTTAGGTTTGG3′. The mice were bred into a C57Bl/6 background; there is no detectable Atxn3 mRNA or protein in the KO mice.

### DNA constructs

GFP-Atxn3 constructs were generated by removing Atxn3-Q22, Atxn3-Q80, Atxn3Q22C14A, and Atxn3Q22UIM^ (all three ubiquitin interacting motifs (UIMs) with serine mutated to alanine) from pFLAG-6A plasmid (provided by Sokol Todi and Henry Paulson) with HindIII and KpnI and ligated into the multiple cloning site of pEGFP-C3 (Clontech Laboratories, Inc). GFP-Atxn3Q22 with UIMs and the catalytic site both mutated was generated by mutating the catalytic cysteine to alanine in the GFP-Atxn3Q22UIM^ construct using the QuickChange Site-Directed Mutagenesis kit (Stratagene). Primers containing the mutation were the following: forward primer, 5′CAA GAA GGC TCA CTT GCT GCT CAA CAT TGC CTG3′; reverse primer, 5′CAG GCA ATG TTG AGC AGC AAG TGA GCC TTC TTG3′ (mutations to change cysteine to alanine are underlined). Reactions and times were performed according to the standard protocol provided in the QuickChange Site-Directed Mutagenesis kit.

**Fig. 1:**
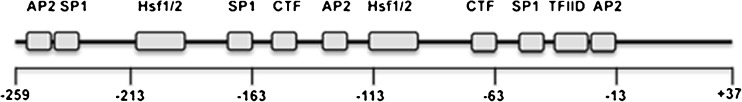
Schematic of 296 base pairs of *HSP1A1* promoter used in the study to drive luciferase. Numbering is relative to the start site and 11 elements involved in basal and stress-induced activity are shown as *boxes* and their transcription factors *above the* *boxes*

The human hsp70 promoter luciferase reporter used in the study had 296 base pairs from −259 to +37 relative to the start site of the human *HSPA1A* gene (Ray et al. 2004; Fig. 1). The promoter reporter was used in mouse fibroblasts, so it is pertinent to provide information and comparison of the similarity of human *HSPA1A* and mouse *Hspa1a* for elements regulating basal- and stress-induced promoter activity (Wu et al. 1986; Greene et al. 1987; Morgan et al. 1987; Williams et al. 1989; Williams and Morimoto 1990; Bevilacqua et al. 1997; Christians et al. 1997; Schug and Overton 1997; Bevilacqua et al. 2000). Nucleotide identity between human and mouse promoters is 70 %. The 11 transcription elements depicted in Fig. 1 and associated transcription factors are the following: two heat shock elements (HSEs; Hsf1/2), three GC boxes (Sp1), three AP2α elements (AP2α), two CCAAT boxes (CTF/CBF/CREB binding protein (CBP)), and one TATA box (TFIID). Of these 11 elements, there are three to four differences between human and mouse: (1) the mouse does not have the GC box at −245 and no obvious element at this position, (2) the mouse replaces the AP2α element at −20 with a GC box, and (3) mouse replaces the CCAAT box at −152 and AP2α element at −137 with a TRE element (AP1 transcription factor) flanked by GC boxes upstream and downstream of the TRE element.

## Results

**Fig. 2:**
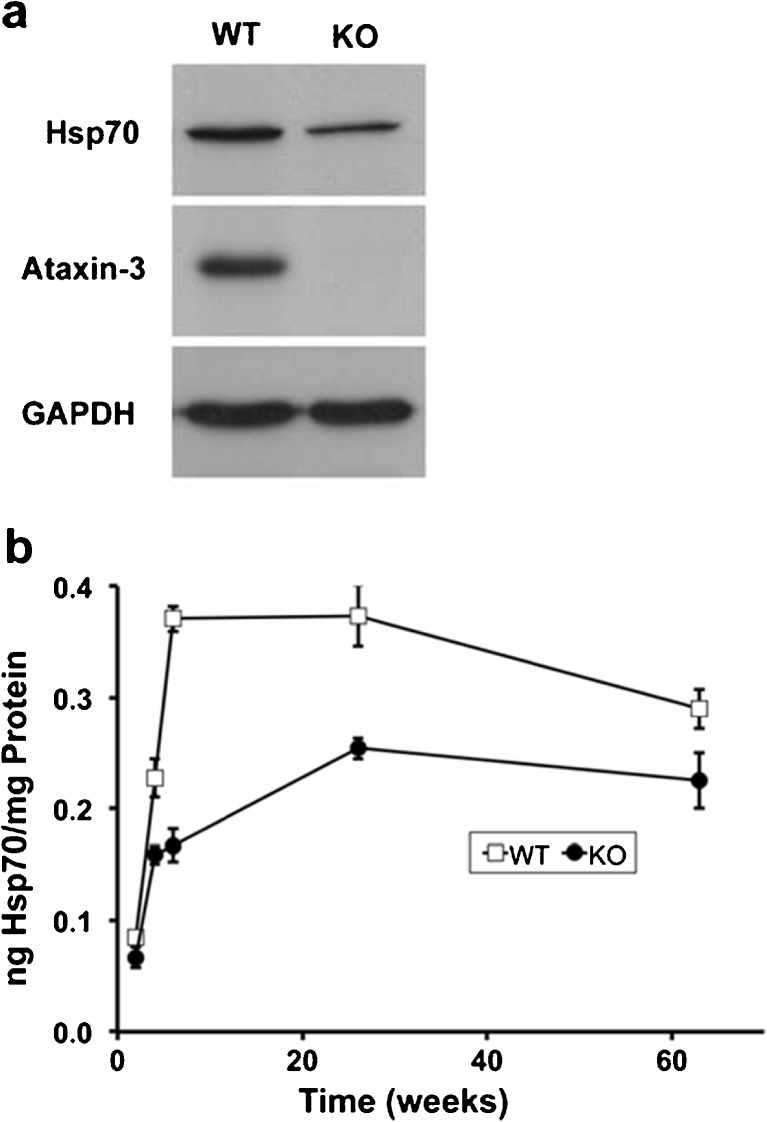
Basal level of hsp70 is decreased in Atxn3 KO brain. **a** Western blot of 6-week-old WT and Atxn3 KO mouse brains. The western blot was probed for hsp70 and then stripped and probed for Atxn3 and GAPDH; GAPDH was used as a loading control. **b** Quantitation of basal levels of hsp70 in WT and Atxn3 KO mouse brain. A mouse hsp70 ELISA was used to quantitate hsp70 in whole brain lysate from mice 2, 4, 6, 26, and 63 weeks of age. Data points represent mean ± SD of three or four WT or KO mice. Hsp70 was significantly lower in KO brains compared to same age WT brains at all ages tested (*P* < 0.05, 2, and 63 weeks; *P* < 0.01, 4, 6,and 26 weeks)

The preliminary observation that hsp70 protein appeared to be lower in Atxn3 KO fibroblasts raised the question: does Atxn3 modulate hsp70 levels and if so, is it a sufficiently robust effect to detect in mouse tissue? To investigate this, we used western blots to examine the level of hsp70 in WT and Atxn3 KO mouse brain (Fig. 2a). Initial western blots indicated that hsp70 protein was lower in KO mouse brain. More extensive experiments using ELISAs to quantitate hsp70 in WT and KO brains at multiple ages suggested that Atxn3-regulated basal hsp70 (Fig. 2b). Levels of hsp70 were significantly lower in KO brain in all ages tested between 2 and 63 weeks (Fig. 2b). By 63 weeks of age, it appeared that WT and KO levels of hsp70 were converging. Levels of hsp70 could not be consistently detected with the ELISA prior to postnatal days 11–12 for KO brain and prior to postnatal days 7–8 for WT brain (not shown).

**Fig. 3:**
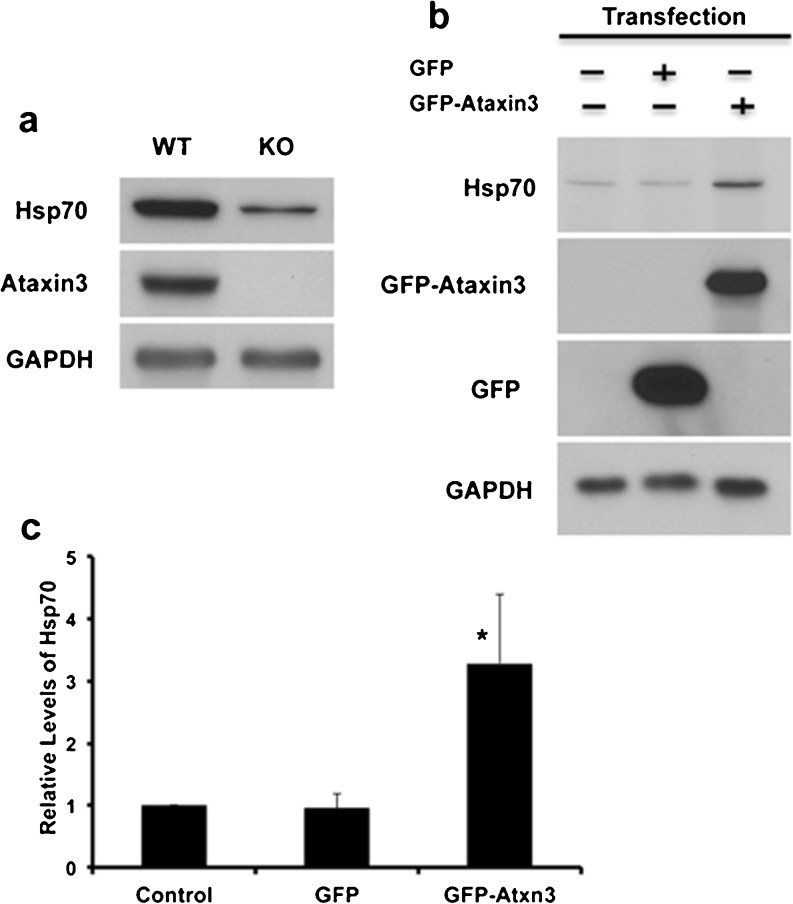
Basal level of hsp70 in cells is regulated by Atxn3. Fibroblast lines were established from WT and Atxn3 KO mice. **a** Western blot of WT and KO cells. GAPDH was used as a loading control. **b** Atxn3 KO cells were transfected with GFP or GFP-Atxn3 and sorted 24 h later to increase the fraction of transfected cells; control KO cells were not transfected. The blot was sequentially probed for hsp70, GAPDH, Atxn3, and GFP. **c** Quantitation of western blots from cell sorting experiments. Data represent the mean ± SD of three independent experiments (*P < 0.05; compared to GFP transfected sorted cells)

Consistent with data from WT and KO mouse brain, the basal level of hsp70 was decreased in Atxn3 KO fibroblast lines (Fig. 3a). To determine if Atxn3 could “rescue” basal level of hsp70 in KO fibroblasts, cells were transfected with GFP or GFP-Atxn3 and underwent cell sorting to increase the fraction of cells expressing GFP or GFP-Atxn3. Transfected GFP-Atxn3 increased the low level of hsp70 in Atxn3 KO cells, while GFP did not alter levels of hsp70 (Fig. 3b, c). Taken together, data in Figs. 2 and 3 were consistent with Atxn3 regulating the basal level of hsp70.

**Fig. 4:**
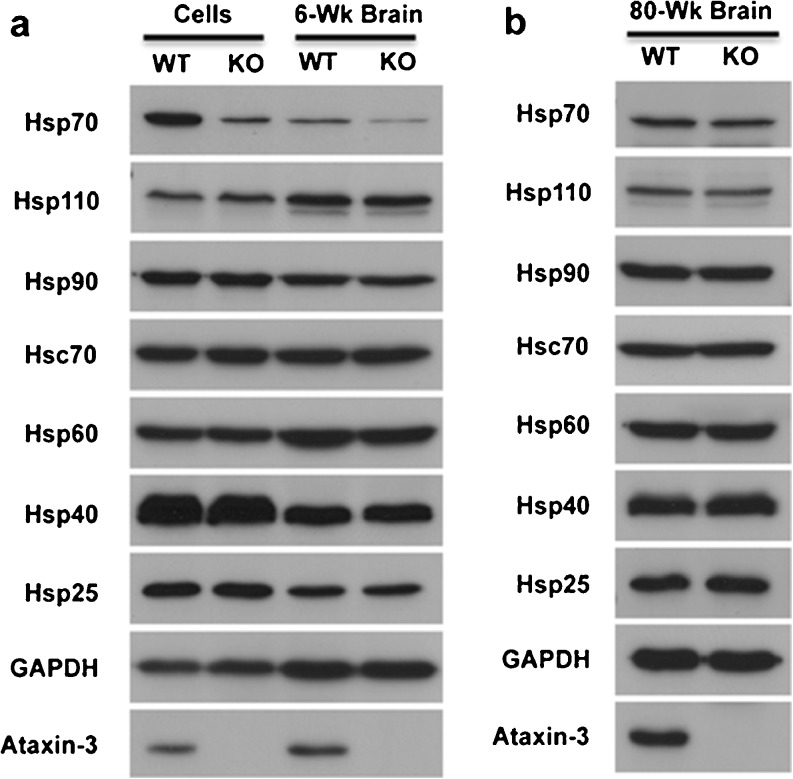
Expression of chaperones in WT and Atxn3 KO brain and fibroblasts. **a** Expression of chaperones in WT and KO fibroblasts and 6-week WT and KO whole brain. Half brains were combined from two different WT and two different KO mice to generate lysates. **b** Expression of chaperones in 80-week WT and KO whole brain. Western blots in panels **a** and **b** were initially probed for hsp70 and then stripped and probed for other proteins. Each chaperone was tested a minimum of three times on different blots and paired with different chaperones on each blot to provide an accurate representation of the composite blot

To determine if Atxn3 regulated other heat shock proteins, western blots of WT and KO mouse brain and cultured fibroblasts were tested for differential expression of representative heat shock proteins from major families: hsp110, hsp90, hsp70, hsc70, hsp60, hsp40/hdj2, and hsp25 (Fig. 4a). In many cases, chaperones had different protein expression levels in fibroblasts compared to brain; however, no chaperone, other than hsp70, showed differential expression between WT and KO cells or brain (Fig. 4a). Although Atxn3 may regulate other chaperones in addition to hsp70, it does not appear that Atxn3 has a general effect on chaperones in fibroblasts or brain. Levels of many chaperones change during aging (Morimoto 2008; Calderwood et al. 2009; Gupta et al. 2010; Anckar and Sistonen 2011; Tower 2011); therefore, we examined the possibility that Atxn3 may modulate levels of chaperones in aging brain. To do so, western blots were generated using 80-week WT and KO mouse brains (Fig. 4b). At 80 weeks of age, WT and KO mouse brain had similar levels of all chaperones, including hsp70. The similar level of hsp70 in WT and KO brain at 80 weeks was consistent with the converging levels of hsp70 in WT and KO brain with increasing age (Fig. 2b).

**Fig. 5:**
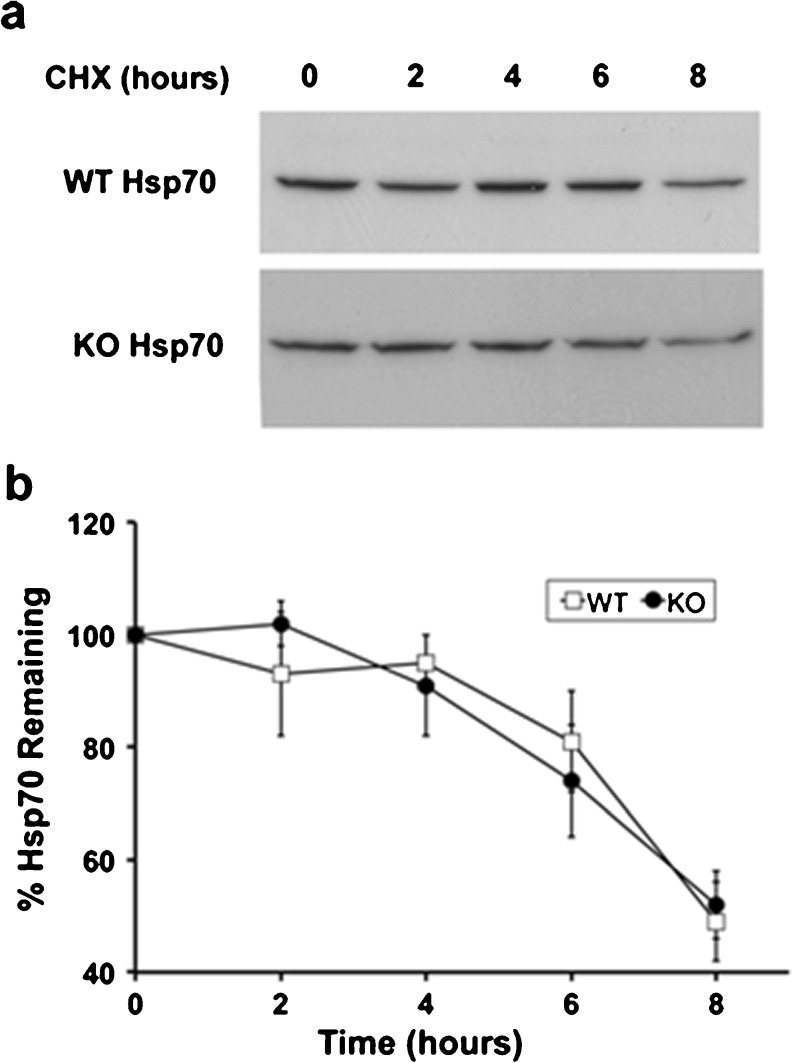
Turnover of hsp70 in WT and Atxn3 KO fibroblasts. **a** Western blot of WT and KO cells treated with cycloheximide (CHX) for 0, 2, 4, 6, or 8 h and probed for hsp70. The KO western blot was exposed for a longer period than the WT blot so that similar bands could be viewed. **b** Quantitation of hsp70 probed western blots. Data represent the mean ± SD of three independent experiments (there is no significant difference between WT and KO)

**Fig. 6:**
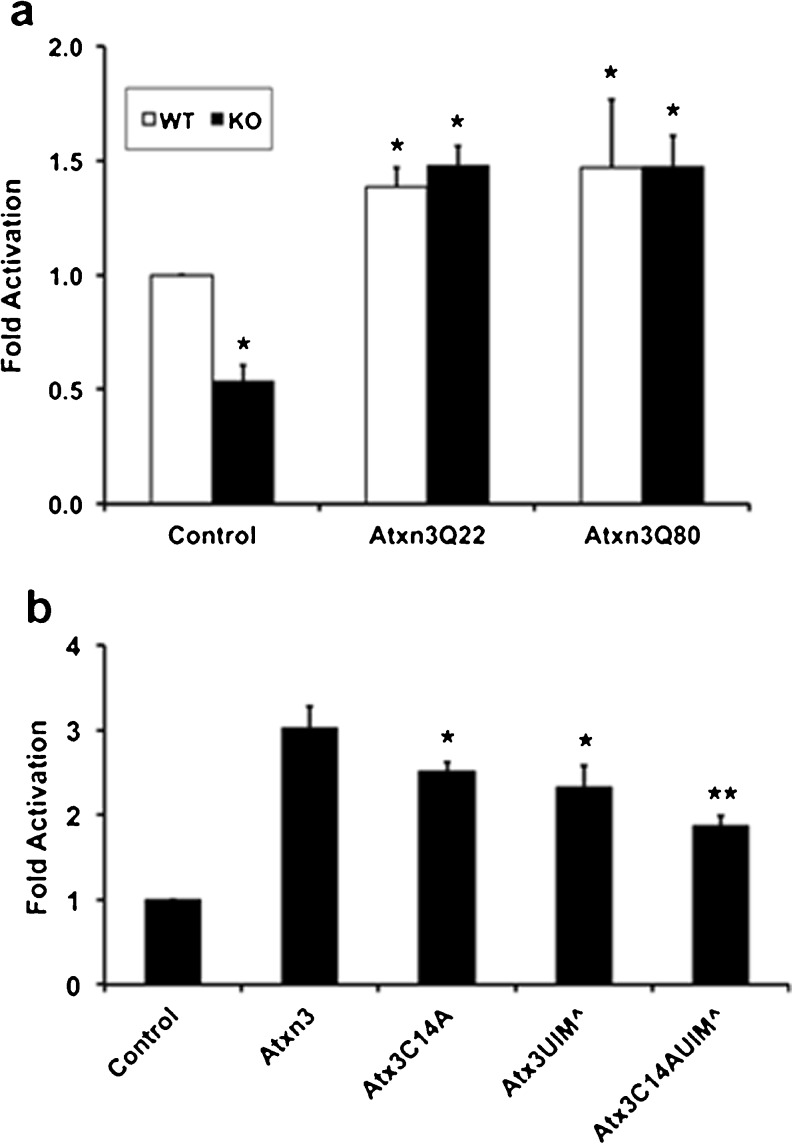
Modulation of basal hsp70 promoter activity by Atxn3. **a** Cells were transfected with hsp70 promoter-luciferase reporter, *Renilla* luciferase, and GFP (control), or GFP-Atxn3Q22, or GFP-Atxn3Q80. Data represent the mean ± SD of three independent experiments with Hsp70 promoter activity expressed relative to WT control cells (*P < 0.05, relative to WT control). **b** Effect of Atxn3 mutations on basal hsp70 promoter activity. KO cells were transfected with hsp70 promoter-luciferase reporter, *Renilla* luciferase, and GFP (control), or GFP-Atxn3Q22, or GFP-Atxn3Q22 with mutations that inactivate DUB activity (Atx3C14A), ubiquitin chain binding (Atx3UIM^), or inactivate both DUB activity and ubiquitin chain binding (Atx3C14AUIM^). Data represent the mean ± SD of three independent experiments with Hsp70 promoter activity expressed relative to control KO cells transfected with GFP (*P < 0.05, **P < 0.025; relative to KO cells transfected with GFP-Atxn3)

Data in Figs. 2, 3, and 4 are consistent with Atxn3 regulating the level of hsp70 in mouse brain and cultured fibroblasts. Atxn3 has multiple cellular functions, including regulating protein degradation and transcriptional regulation. Initially, we determined if hsp70 turnover was altered in mouse Atxn3 KO cells. Treatment of WT and KO cells with 10 uM cycloheximide indicated that turnover of hsp70 was very similar in WT and KO fibroblasts (Fig. 5a, b). We then investigated the possibility that Atxn3 regulated the hsp70 promoter. Atxn3 can function as either a repressor or enhancer (Li et al. 2002; Evert et al. 2006; Araujo et al. 2011). Hsp70 promoter activity was monitored using an hsp70 promoter-luciferase reporter from the human *HSPA1A* gene (Ray et al. 2004; see Fig. 1). Basal hsp70 promoter reporter activity was approximately two times higher in WT compared to KO cells (control, Fig. 6a). Transfecting GFP did not alter the level of hsp70 promoter luciferase (not shown). Transfecting WT and KO cells with Atxn3 increased promoter activity to similar levels of activation (Fig. 6a). This resulted in ∼50 % increase in promoter activity in WT cells and ∼200 % increase in KO cells. Pathogenic Atxn3Q80 with an expanded tract of 80 glutamines was equally effective as wild-type Atxn3Q22 increasing basal promoter activity in both WT and KO cells (Fig. 6a). However, mutations that altered DUB activity or ability to bind ubiquitin chains were less effective in restoring promoter activity (Fig. 6b). Atxn3 KO fibroblasts were transfected with hsp70 luciferase reporter, *Renilla* luciferase, and wild-type Atxn3 or Atxn3 with mutations that inhibited DUB activity (Atxn3C14A), or binding ubiquitin chains (Atxn3UIM^), or mutations for both DUB activity and binding ubiquitin chains (Atxn3C14AUIM^). All mutations significantly decreased the ability of Atxn3 to “rescue” basal promoter activity (Fig. 6b). However, Atxn3 with both DUB activity and ubiquitin chain binding mutated still had about 50 % increase in promoter activity indicating that other functions of Atxn3 are involved in modulating basal hsp70 promoter activity.

**Fig. 7:**
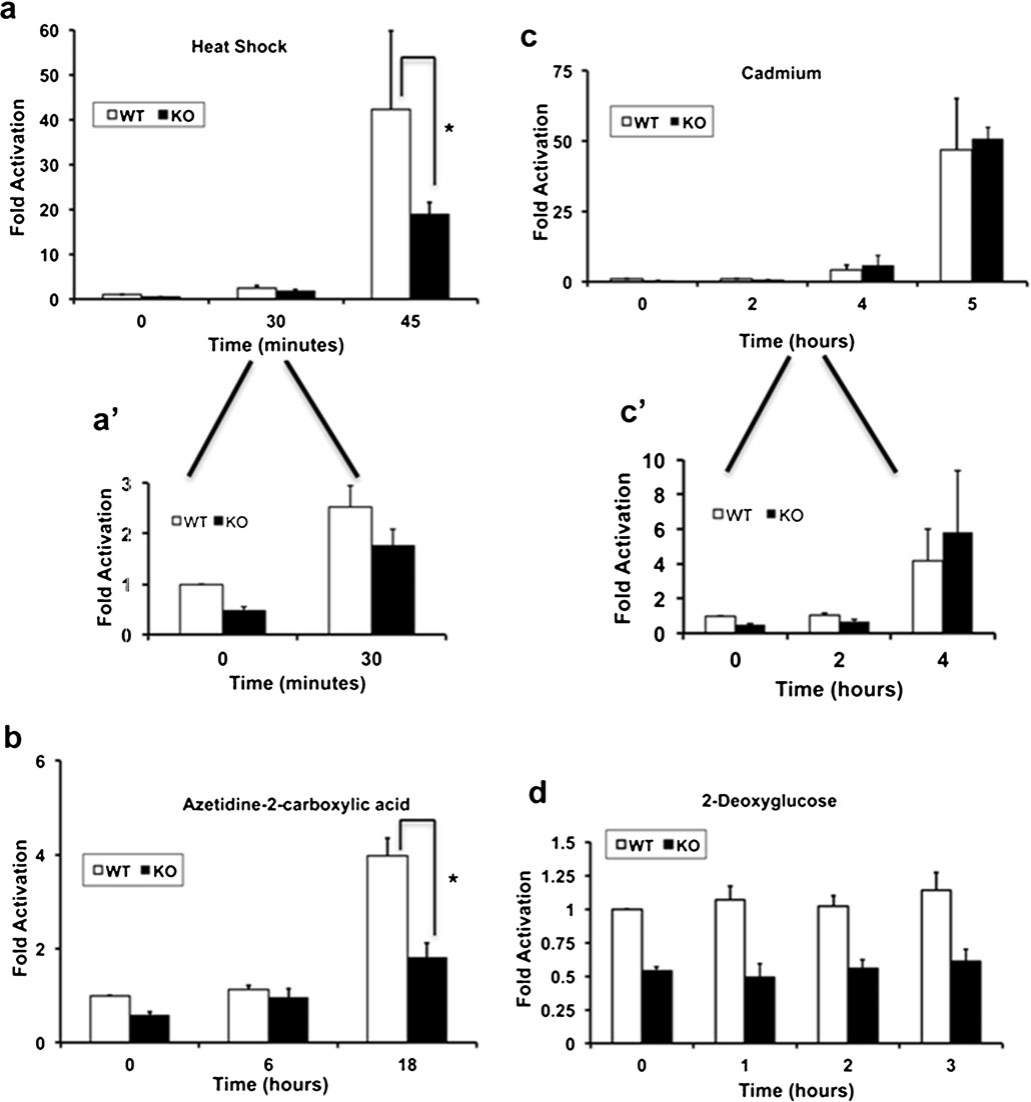
Stress-induced hsp70 promoter activity in WT and Atxn3 KO cells. WT and KO cells were transfected with hsp70 promoter-luciferase reporter and *Renilla* luciferase. After 24 h cells were exposed to: **a** 42 °C heat shock, **b** 2.5 mM azetidine-2-carboxylic acid, **c** 50 uM cadmium, or **d** 10 mM 2-deoxyglucose; at the indicated times cells were lysed and lucifierase activity measured immediately. Panels *a′* and *c*′ are enlarged areas of early time points for **a** and **c**. Hsp70 promoter activity is expressed relative to WT control cells (0 time). Data represent the mean ± SD of three or four independent experiments (*P < 0.05; comparison of WT and KO cells responding to stress; all basal levels and all two-DG times were significantly different between WT and KO cells but are not identified with *asterisks*)

To determine if Atxn3 modulation of hsp70 promoter activity extended beyond basal activity, WT and KO cells were treated with stressors that induce hsp70 (heat shock, cadmium, and AZE), as well as a stressor that typically does not induce hsp70, 2-DG. Following heat shock, there was a striking increase in hsp70 promoter activity between 30 and 45 min (Fig. 7a), while hsp70 reporter activity increased between 6 and 18 h in AZE-treated cells (Fig. 7b) and between 2 and 5 h for cadmium-treated cells (Fig. 7c). Hsp70 promoter activity was higher in WT cells in response to heat shock and AZE. This suggested that Atxn3 modulated stress-induced hsp70 promoter activity in response to these stresses. In contrast, following cadmium stress hsp70 promoter activity had similar activity in WT and KO cells at 5 h (Fig. 7c). As expected, 2-DG had no obvious effect on hsp70 promoter activity in either WT or KO cells. An interesting aspect of the data was that at early times hsp70 promoter activity increased more rapidly in KO cells compared to WT cells even with stresses that ultimately had a more robust induction in WT cells (Fig. 7 a', b, c'). Comparing KO to WT cells as a ratio of hsp70 promoter induction (KO/WT) showed a more rapid increase early in KO cells and a more rapid increase later in WT cells: (a) heat shock: 0–30 min = 1.8, 30–45 min = 0.62; (b) AZE: 0–6 h = 4.04, 6–18 h = 0.35; (c) cadmium: 0–2 h = 10.0, 2–4 h = 2.56, 0–4 h = 3.54, 4–5 h = 0.84.

**Fig. 8:**
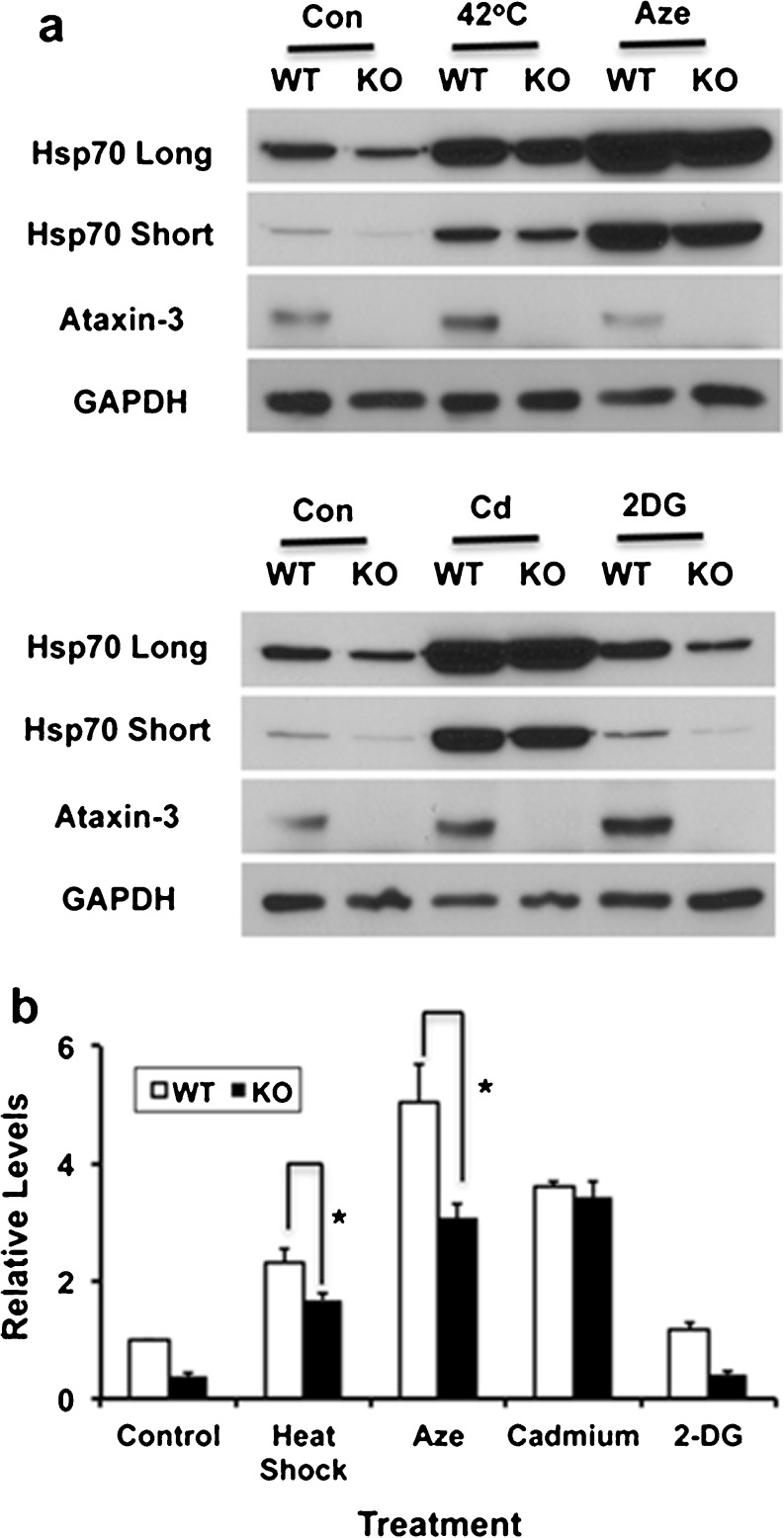
Stress-induced hsp70 in WT and Atxn3 KO cells. **a** Western blots of WT and KO cells exposed to no stress (Con), heat shock for 45 min (42 °C), 2.5 mM AZE for 18 h, 50 uM cadmium for 5 h (Cd) or 10 mM 2-DG for 2 h. Long and short exposures of hsp70 are included to cover the wide range of hsp70 levels. GAPDH is a loading control. **b** Quantitation of western blots. Data represent the mean ± SD of three independent experiments (*P < 0.05; comparison of WT and KO cells responding to stress)

Atxn3 appeared to modulate hsp70 promoter activity in response to heat shock and AZE stress but not to cadmium stress. To determine if hsp70 protein levels showed similar changes as hsp70 promoter activity in response to stress, cells were treated with 42 °C heat (45 min), 2.5 mM AZE (18 h), 50 uM cadmium (5 h), or 10 mM 2-DG (2 h) and examined using western blots. Times of exposure to stressors were chosen to coincide with robust promoter activity (see Fig. 7). Hsp70 protein increased following all stresses except 2DG (Fig. 8a, b). Both heat- and AZE-treated KO cells had lower levels of hsp70 compared to WT cells (Fig. 8a,b); this was consistent with decreased hsp70 promoter activity in KO versus WT cells at the same times (Fig. 7a, b). Hsp70 protein was very similar in WT and KO cells following cadmium exposure (Fig. 8a, b) consistent with similar levels of hsp70 promoter activity after 5 h of exposure (Fig. 7c).

**Fig. 9:**
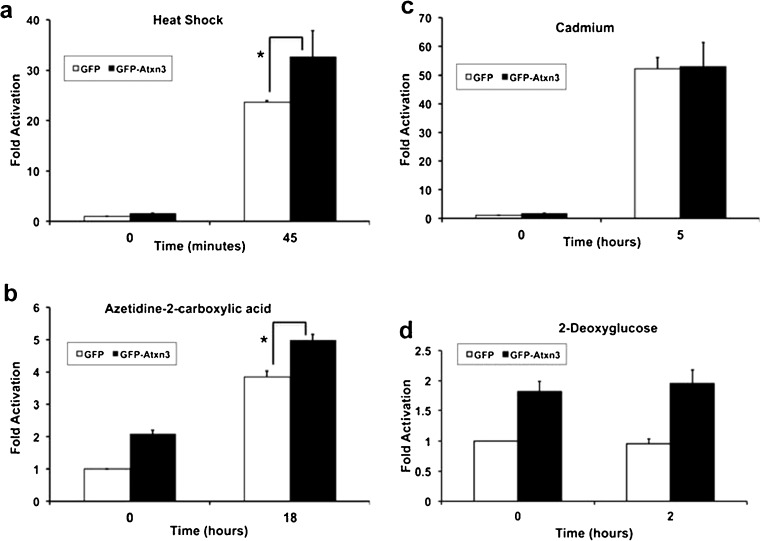
Atxn3 modulates stress-induced hsp70 promoter activity in Atxn3 KO cells. KO cells were transfected with hsp70 promoter-luciferase reporter, *Renilla* luciferase, and either GFP or GFP-Atxn3Q22 and exposed to no stress (0 time), **a** 42 °C heat shock for 45 min, **b** 2.5 mM azetidine-2-carboxylic acid for 18 h, **c** 50 uM cadmium for 5 h, or **d** 10 mM 2-deoxyglucose for 2 h. Hsp70 promoter activity is expressed relative to GFP control cells (0 time) and data represent the mean ± SD of three independent experiments (*P < 0.05; comparison of WT and KO cells responding to stress)

Data generated using WT and KO fibroblasts were consistent with Atxn3 increasing both hsp70 promoter activity and hsp70 protein in response to heat shock and AZE stress, while Atxn3 had little or no effect on cadmium stress (Figs. 7 and 8). To determine if replacing Atxn3 in KO cells also showed similar effects of modulating stress-induced hsp70 promoter activity KO fibroblasts were transfected with either GFP or GFP-Atxn3. Under basal conditions (0 time), transfected Atxn3 increased basal activity of the hsp70 promoter as previously shown in Fig. 6. In response to AZE and heat stress, KO cells transfected with GFP-Atxn3 had increased hsp70 promoter activity compared to KO cells transfected with GFP (Fig. 9a, b). In contrast, GFP and GFP-Atxn3 had similar effects with cadmium stress, suggesting that Atxn3 was not modulating cadmium-induced hsp70 promoter activity; this is consistent with data in Fig. 7c using WT and KO cells. As expected, 2DG-treated KO cells were very similar to control cells with Atxn3 increasing basal hsp70 promoter activity and a similar increase in hsp70 promoter activity in 2DG-treated cells (Fig. 9d).

**Fig. 10:**
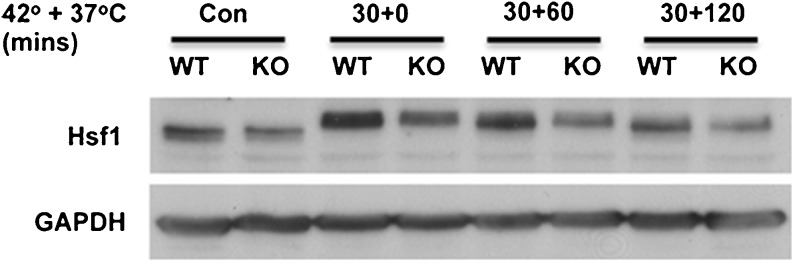
Hsf1 in WT and KO fibroblasts under basal conditions and heat shock stress. Western blot of WT and KO cells maintained at 37 °C (Con) or exposed for 30 min at 42 °C and recovered at 37 °C for 0 min (30 + 0), 60 min (30 + 60), or 120 min (30 + 120) and probed for Hsf1. GAPDH is a loading control

**Fig. 11:**
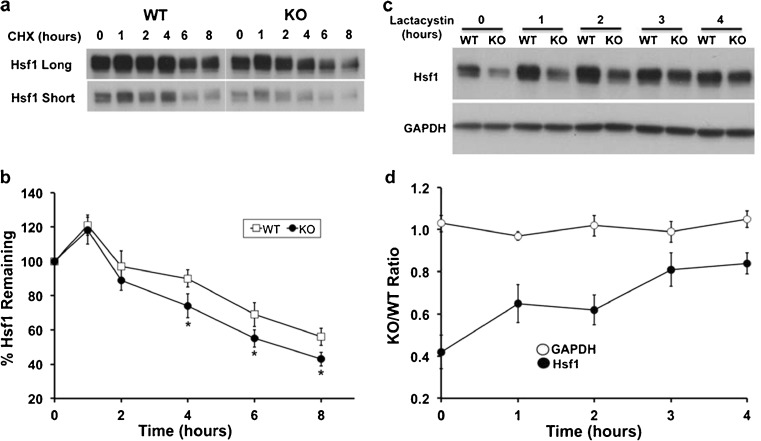
Turnover of Hsf1 in WT and Atxn3 KO fibroblasts. **a** Western blots of WT and KO cells treated with 10 uM cycloheximide (CHX) for 0, 1, 2, 4, 6, or 8 h and probed for Hsf1. Long and short exposures of Hsf1 are included to cover the wide range of Hsf1 protein. **b** Quantitation of western blots of CHX-treated cells probed with Hsf1. Data represent the mean ± SD of three independent experiments (*P < 0.05; comparison of WT and KO cells at same time point). **c** Western blots of WT and KO cells treated with 5 uM lactacystin for 0, 1, 2, 3, or 4 h and probed for Hsf1 or GAPDH. **d** Data are presented as KO/WT ratios from western blots of lactocystin-treated cells and probed with Hsf1 or GAPDH. Data represent the mean ± range bars of two independent experiments

Replacing Atxn3 in KO cells increased hsp70 promoter activity following heat shock and AZE stress. Based on known functions of Atxn3, it could have direct effects on transcription or effects through the ubiquitin–proteasome pathway by altering key proteins that regulate hsp70 induction in response to stress. Initial experiments focused on Hsf1. Hyperphosphorylation associated with activation of Hsf1 had similar temporal shifts in WT and KO cells in response to heat shock; however, the level of Hsf1 was consistently decreased in KO cells under basal conditions and in response to heat shock stress (Fig. 10). WT and KO fibroblasts were treated with cycloheximide to determine if Hsf1 had altered turnover in KO cells. Turnover of Hsf1 was more rapid in KO cells compared to WT cells (Fig. 11a, b). Treating cells with the proteasome inhibitor, clasto-lactacystin-β-lactone, increased the level of Hsf1 (Fig. 11c). However, Hsf1 in KO cells accumulated more rapidly relative to WT cells while GAPDH accumulation was similar in WT and KO cells (Fig. 11d). This is consistent with the more rapid turnover of Hsf1 in KO cells (Fig. 11a, b).

## Discussion

Hsp70 maintains homeostasis under basal conditions by regulating multiple processes. These processes include folding newly translated proteins, binding aberrant proteins to protect cellular proteins from nonproductive or “toxic” interactions, as well as facilitating their degradation (Sherman and Goldberg 2001; Hartl and Hayer-Hartl 2002; Bukau et al. 2006). Under stressful conditions, hsp70 increases rapidly and in concert with protein degradation pathways protect cells from aberrant and misfolded proteins. Data in the current study are consistent with Atxn3 modulating levels of hsp70 and previous studies are consistent with Atx3 regulating protein degradation (Zhong and Pittman 2006; Wang et al. 2006; Schmitt et al. 2007; Winborn et al. 2008; Durcan et al. 2010; Scaglione et al. 2011) suggesting that Atxn3 helps regulate two key cellular processes that protect cells against aberrant proteins.

Maintaining the basal level of hsp70 is critical for normal functions including protein folding (Hartl and Hayer-Hartl 2002), cell proliferation (Milarski and Morimoto 1986; Feder et al. 1992; Krebs and Feder 1997; Volloch and Sherman 1999), apoptosis (Vayssier and Polla 1998; Li et al. 2000; Beere and Green 2001), and potentially helping maintain Hsf1 in a nonfunctional state in the absence of stress (Nollen and Morimoto 2002). Basal levels of hsp70 vary depending on type of cell, phenotype, tissue, or individual humans (Greene et al. 1987; Locke et al. 1991; Cowley et al. 1995; Muller et al. 1996; Manzerra et al. 1997; D’Souza and Brown 1998; Xiao et al. 2003; O’Neill and Noble 2004; Noonan et al. 2007; Salway et al. 2011). It is not clear how the basal level of hsp70 is established and maintained; however, Atxn3 is a widely expressed protein and data in this study is consistent with Atxn3 helping maintain levels of hsp70 in mouse brain and fibroblasts by modulating hsp70 promoter activity.

The hsp70 promoter has multiple elements that maintain and modulate the basal level of hsp70 (Fig. 1). Having multiple basal elements should allow the hsp70 promoter to respond to variable changes in the local environment independent of modulation of hsp70 through the stress-induced pathway. The hsp70 promoter reporter used in this study consisted of 296 base pairs from −259 to +37 relative to the start site of the human *HSPA1A* gene (Ray et al. 2004); this encompasses the area of the hsp70 promoter that contains basal and stress-induced hsp70 regulatory elements. It includes proximal and distal HSE for stress-induced activity as well as nine elements that can contribute to hsp70 basal activity including three GC boxes, two CCAAT boxes, three AP2α elements, and a TATA box (Wu et al. 1986; Greene et al. 1987; Morgan et al. 1987; Williams et al. 1989; Williams and Morimoto 1990; Bevilacqua et al. 1997; Christians et al. 1997). The mouse *Hspa1a* promoter is similar to the human promoter with proximal and distal HSEs, four GC boxes, one CCAAT box, one AP2α element, one AP1 element, and a TATA box (Bevilacqua et al. 1997; Christians et al. 1997; Schug and Overton 1997; Bevilacqua et al. 2000).

Atxn3 can bind directly to DNA; however, it is more common for Atxn3 to interact with transcription factors, co-repressors, or co-activators and modulate their functions (Li et al. 2002; Evert et al. 2006; Araujo et al. 2011). The initial study by Li et al. (2002) showed that Atxn3 interacted with three major histone acetyltransferases, CBP, p300, and p300/CBP-associated factor and inhibited their acetyltransferase activity resulting in repression of model gene promoters. p300 is associated with transcriptional complexes on many promoters including complexes on the HSE and CCAAT boxes in the hsp70 promoter (Li et al. 1998; Xu et al. 2008; Westerheide et al. 2009). In addition to binding and altering activity of histone acetyltransferases Atxn3 can bind to DNA and recruit histone deacetylase 3 and nuclear receptor corepressor, NCoR, to repress transcription of the matrix metalloproteinase-2 promoter (MMP2; Evert et al. 2006). Atxn3 UIMs were required to repress MMP2 transcription but Atxn3 catalytic DUB activity was not required. A recent study showed that in response to oxidative stress Atxn3 interacted with the transcription factor, FOXO4, and enhanced activation of manganese superoxide dismutase (SOD2) (Araujo et al. 2011). Other experiments indicated that shRNA knockdown of endogenous Atnx3 decreased basal expression of SOD2. This study showed that Atxn3 had an important role in responding to oxidative stress as well as maintaining the basal level of SOD2 (Araujo et al. 2011).

Using tissue and cells from WT and Atxn3 KO mice, we showed that basal levels of hsp70 protein and hsp70 promoter activity were lower in KO brain and cells (Figs. 2, 3a, 4, and 6a). Replacing Atxn3 in KO cells increased the low level of hsp70 protein and promoter activity (Figs. 3b, c and 6). Mutating the catalytic DUB function or UIMs in Atxn3 diminished its ability to increase basal hsp70 promoter activity (Fig. 6b). Loss of Atxn3 decreased the level of hsp70 protein; however, it did not appear to have appreciable effects on protein levels of 6 other chaperones tested in cells or brain (Fig. 4).

Although Atxn3 regulated hsp70 basal promoter activity and protein, it was unexpected that Atxn3 also modulated stress-induced hsp70 promoter activity and protein following heat shock and AZE stresses. Hsp70 basal promoter elements modulate HSE; the increased activity from basal elements is greater than a simple additive effect (Williams and Morimoto 1990; Imbriano et al. 2001). The working model for basal promoter elements modulating the stress-induced promoter is that a subset of protein complexes assembled on hsp70 basal promoter elements also interact with proteins in complexes associated with the HSE and either enhance or decrease HSE transcription. Williams and Morimoto (1990) showed that basal promoter activity was important for robust stress-induced HSE promoter activity. Mutations in GC and CCAAT boxes that decreased basal activity also decreased stress-induced promoter activity. Mutations in basal elements that did not affect basal activity did not alter stress-induced promoter activity (Williams and Morimoto 1990). Experiments by Imbriano et al. (2001) were consistent with the CCAAT-box binding transcription factors, CTF and NF1, modulating both basal and stress-induced hsp70 activity by binding to the CCAAT box and interacting with HSF1 bound to HSE.

Basal promoter activity was lower in Atxn3 KO cells and increased when Atxn3 was replaced (Fig. 6b); however, a much greater increase in promoter activity occurred when Atxn3 was replaced in KO cells that were exposed to heat shock stress (Fig. 9a). Atxn3 increased hsp70 basal promoter activity and this may be responsible for some of the enhanced stress-induced promoter activity; however, Atxn3 may modulate stress-induced activity through other mechanisms. Atxn3 binds to p300 and inhibits its acetyltransferase activity (Li et al. 2002). p300 is recruited to the Hsf1 complex assembled on the HSE promoter in response to heat shock and acetylates Hsf1 (Xu et al. 2008; Westerheide et al. 2009). Acetylation of Hsf1 decreases its binding to DNA resulting in decreased promoter activity (Westerheide et al. 2009; Anckar and Sistonen 2011). Therefore, Atxn3 may interact with p300 bound to the HSE transcription complex and inhibit p300 acetyltransferase activity, resulting in increased Hsf1 stress-induced activity. Alternatively, Atxn3 functions in the ubiquitin proteasome pathway (Zhong and Pittman 2006; Wang et al. 2006; Schmitt et al. 2007; Durcan et al. 2010; Scaglione et al. 2011) and data in Figs. 10 and 11 suggest that Atxn3 increases the level of Hsf1 protein. The lower level of Hsf1 in KO cells may reflect an increased turnover of Hsf1 in the absence of Atxn3 (Fig. 11). Altered turnover and a more rapid accumulation of Hsf1 in KO cells treated with a proteasome inhibitor are consistent with Atxn3 regulating Hsf1 protein through the ubiquitin proteasome pathway (Fig. 11).

Prior to identifying Atxn3 as a DUB, several studies were consistent with Atxn3 being involved in the ubiquitin proteasome pathway. Atxn3 had ubiquitin interacting motifs (Hofmann and Falquet 2001) and interacted with VCP/p97, HR23B, and proteasomes (Wang et al. 2000; Doss-Pepe et al. 2003). Once Atxn3 was identified as a DUB (Burnett et al. 2003; Scheel et al. 2003), several studies suggested that Atxn3 functions in cellular proteostasis by decreasing degradation of a model protein in cells (Burnett et al. 2003), regulating endoplasmic reticulum degradation (Wang et al. 2006; Zhong and Pittman 2006), and showing that KO Atxn3 mice had increased levels of ubiquitinated proteins (Schmitt et al. 2007). In addition, Atxn3 protected *Drosophila* exposed to aggregated forms of huntingtin, ataxin-1, and Atxn3 but did not protect against tau toxicity or a dominant negative hsp70 (hsp70K71E; Warrick et al. 2005).

Recent studies indicate that Atxn3 not only functions in routine maintenance of cellular proteostasis, but also responds to stressful conditions. Reina et al. (2010) showed that Atxn3 rapidly localized to the nucleus in response to heat shock and oxidative stress in an Hsf1-independent mechanism and Atxn3 protected cells from toxic heat shock, but not from toxic ER stress or proteasome inhibition. A recent study by Araujo et al. (2011) showed that Atxn3 translocated to the nucleus in response to oxidative stress and in combination with the transcription factor, FOXO4, increased promoter activity and expression of SOD2. Initial observations of Atxn3 KO in *Caenorhabditis elegans* did not detect any obvious effects (Rodrigues et al. 2007); however, more recently, this group determined that Atxn3 regulated the stress response in *C. elegans* (Rodrigues et al. 2011). Surprisingly, Atxn3 KO *C. elegans* were more resistant to toxic heat stress and several chaperones including members of the hsp70 family were increased. The protective effects were independent of Hsf1 but required the DAF-16 pathway; parallel experiments by Kuhlbrodt et al. (2011) showed that the double knockout of Atxn3 and CDC48/p97 (a protein that shuttles ubiquitinated proteins to proteasomes) increased lifespan in *C. elegans* through the DAF-16 pathway. DAF-16 is a FOXO family transcription factor that controls longevity, metabolism and select developmental processes in *C. elegans* (Lee et al. 2003). Mammalian orthologs of DAF-16 are FOXO1,3, and 4. It is notable that Atxn3 interacts with mammalian FOXO4 and activates basal and stress-induced expression of SOD2 (Araujo et al. 2011); this is opposite in *C. elegans* where Atxn3 KO exhibits enhanced transcription of *sod-3* through DAF-16 (Rodrigues et al. 2011). Differences between *C. elegans* and mammals in modulating chaperones may be as simple as Atxn3 functioning as a co-repressor or a co-activator at different genes; however, regulation and response to stress in mammals and *C. elegans* are very likely more complex.

Atxn3 responds to stress, functions in cellular proteostasis, and modulates hsp70. Hsp70 is a critical protein for cellular homeostasis; however, is hsp70 an important aspect of Atxn3 functions and response to stress? It is interesting that hsp70 promoter activity and protein were lower in Atxn3 KO cells in response to heat and AZE stress while cadmium stress had similar effects in WT and KO cells (Figs. 7, 8, and 9). Early effectors of stress signaling are different between cadmium and heat shock/AZE stresses. An increase in misfolded and aberrant proteins initiate stress responses in cells exposed to heat shock and AZE (Morimoto 1998) while cadmium initiates a stress response by releasing ER calcium, generating reactive oxygen species and activating MAP kinase signaling pathways (Thevenod 2009). In that Atxn3 has several functions associated with misfolded proteins including ER protein degradation (Wang et al. 2006; Zhong and Pittman 2006), protecting against excess misfolded protein by forming aggresomes (Burnett and Pittman 2005; Ouyang et al. 2012) and protecting organisms from misfolded and aggregated protein (Warrick et al. 2005) indicates that Atxn3 responds to misfolded proteins. Therefore, it seems that Atxn3 would most likely respond to stresses like heat shock and AZE associated with excess misfolded proteins. In the context of excess misfolded proteins, hsp70 may be an important aspect of Atxn3 protective effects (Warrick et al. 1999, 2005). Atxn3 is expressed widely in plants and animals and present in most or all tissues in humans and mice. Therefore, Atxn3 may be a general modulator of basal hsp70 in many species and tissues as well as being a potential modulator of hsp70 in response to stresses associated with excess misfolded proteins.

## Footnotes

1 Hsp70 refers to both human and mouse orthologous genes (*HSPA1A* and *Hspa1a*) and proteins (HSPA1A and hspa1a, b)
